# Supplementary material for: The Impact of the COVID-19 Pandemic on Mental Health, Occupational Functioning, and Professional Retention Among Health Care Workers and First Responders
Source: J Gen Intern Med. 2021 Dec 16;37(2):397–408. doi: 10.1007/s11606-021-07252-z (PMC8675543; doi:10.1007/s11606-021-07252-z)
Supplement: Supplementary file 1 — Supplementary file1 (DOCX 1114 KB) [file 11606_2021_7252_MOESM1_ESM.docx]

Supplemental Tables:

Supplementary Table 1: Items used to assess occupational functioning and expected retention in chosen field and associated response frequencies across participant groups.

|  | | Health Care Workers | | | First Responders | | | |
| --- | --- | --- | --- | --- | --- | --- | --- | --- |
|  | | All HCW | Physicians | Nurses | All FR | LEO and Fire | EMS |  |
|  |  | N=255 | N=56 | N=159 | N=169 | N=64 | N=97 |  |
| How likely do you think it is that you will still be working in your current field in 5-10years | not at all likely | 14.9% | 8.9% | 20.1% | 17.2% | 17.2% | 16.5% |  |
|  | a little likely | 20.0% | 10.7% | 20.8% | 17.2% | 14.1% | 19.6% |  |
|  | moderately likely | 23.9% | 23.2% | 27.0% | 27.2% | 25.0% | 27.8% |  |
|  | highly likely | 41.2% | 57.1% | 32.1% | 38.5% | 43.8% | 36.1% |  |
|  |  | N=260 | N=56 | N=161 | N=170 | N=64 | N=98 |  |
| How have your experiences providing care during the  COVID-19 pandemic affected your interest, willingness,  or ability to continue working in your current field? | significantly decreased | 22.3% | 14.3% | 27.3% | 14.7% | 14.1% | 15.3% |  |
|  | somewhat decreased | 32.7% | 32.1% | 31.7% | 25.9% | 21.9% | 28.6% |  |
|  | no change | 31.2% | 41.1% | 26.7% | 44.7% | 46.9% | 43.9% |  |
|  | somewhat increased | 9.2% | 10.7% | 8.1% | 9.4% | 9.4% | 9.2% |  |
|  | significantly increased | 4.6% | 1.8% | 6.2% | 5.3% | 7.8% | 3.1% |  |
|  |  | N=259 | N=56 | N=160 | N=170 | N=64 | N=98 |  |
| I have trouble doing all of my usual work | Never | 13.1% | 12.5% | 13.8% | 22.9% | 32.8% | 17.3% |  |
|  | Rarely | 30.5% | 33.9% | 27.5% | 34.1% | 32.8% | 35.7% |  |
|  | Sometimes | 38.2% | 37.5% | 40.0% | 33.5% | 29.7% | 36.7% |  |
|  | Usually | 12.4% | 10.7% | 13.1% | 7.6% | 3.1% | 9.2% |  |
|  | Always | 5.8% | 5.4% | 5.6% | 1.8% | 1.6% | 1.0% |  |
|  |  | N=259 | N=56 | N=159 | N=168 | N=63 | N=98 |  |
| I have trouble doing all the work that is really important to me | Never | 15.4% | 14.3% | 17.0% | 23.2% | 28.6% | 20.4% |  |
|  | Rarely | 28.2% | 26.8% | 30.8% | 26.8% | 30.2% | 24.5% |  |
|  | Sometimes | 35.1% | 41.1% | 30.2% | 35.7% | 25.4% | 42.9% |  |
|  | Usually | 15.8% | 14.3% | 16.4% | 10.7% | 14.3% | 7.1% |  |
|  | Always | 5.4% | 3.6% | 5.7% | 3.6% | 1.6% | 5.1% |  |
|  |  | N=218 | N=51 | N=133 | N=137 | N=53 | N=78 |  |
| Thoughts that you would be better off dead, or of hurting yourself | Not at all | 87.6% | 96.1% | 84.2% | 81.0% | 86.8% | 75.6% |  |
|  | Several Days | 7.8% | 3.9% | 9.8% | 12.4% | 5.7% | 17.9% |  |
|  | More than half the days | 4.1% | 0.0% | 6.0% | 2.9% | 3.8% | 2.6% |  |
|  | Nearly every day | 0.5% | 0.0% | 0.0% | 3.6% | 3.8% | 3.8% |  |

Supplementary Table 2: Characterization of missing data. Each column represents an analysis of all available data for either all participants (“All Available”) or for all participants who have completed all items of a particular instrument. Statistically significant differences between respondents who do versus do not have data for a specific instrument are indicated (*p<.05, **p<.01, ***p<.001, without correction for multiple comparisons).

|  | All Available | Complete ISI | Complete CROS | Complete work assessment | Complete occupational retention assessment | Complete PCL5 | Complete GAD7 | Complete PHQ9 |
| --- | --- | --- | --- | --- | --- | --- | --- | --- |
| N | 510 | 496 | 444 | 433 | 429 | 402 | 367 | 357 |
| Age | 41.29±10.82 | 41.29±10.82 | 41.32±10.86 | 41.20±10.78* | 41.34±10.81 | 41.23±10.82 | 41.26±10.85 | 41.14±10.75 |
| Gender:F | 268/362 (74.03%) | 268/362 (74.03%) | 264/358 (73.74%) | 267/360 (74.17%) | 264/358 (73.74%) | 265/359 (73.82%) | 263/357 (73.67%) | 261/355 (73.52%) |
| Married | 208/360 (57.78%) | 208/360 (57.78%) | 205/356 (57.58%) | 207/358 (57.82%) | 207/356 (58.15%) | 208/357 (58.26%) | 206/355 (58.03%) | 207/353 (58.64%)* |
| Veteran | 33/362 (9.12%) | 33/362 (9.12%) | 33/358 (9.22%) | 33/360 (9.17%) | 33/358 (9.22%) | 33/359 (9.19%) | 33/357 (9.24%) | 32/355 (9.01%) |
| FR | 200/510 (39.22%) | 195/496 (39.31%) | 173/444 (38.96%) | 168/433 (38.8%) | 169/429 (39.39%) | 154/402 (38.31%) | 139/367 (37.87%) | 137/357 (38.38%) |
| LEO+fire | 73/510 (14.31%) | 72/496 (14.52%) | 65/444 (14.64%) | 63/433 (14.55%) | 64/429 (14.92%) | 61/402 (15.17%) | 54/367 (14.71%) | 53/357 (14.85%) |
| Physician | 60/510 (11.76%) | 59/496 (11.9%) | 55/444 (12.39%) | 56/433 (12.93%) | 56/429 (13.05%)* | 53/402 (13.18%) | 51/367 (13.9%)* | 51/357 (14.29%)** |
| Had covid | 160/449 (35.63%) | 160/449 (35.63%) | 158/443 (35.67%) | 155/432 (35.88%) | 154/428 (35.98%) | 137/401 (34.16%) | 120/366 (32.79%)* | 118/356 (33.15%)* |
| Family member Covid | 136/450 (30.22%) | 136/450 (30.22%) | 135/444 (30.41%) | 131/433 (30.25%) | 130/429 (30.3%) | 117/402 (29.1%) | 105/367 (28.61%) | 104/357 (29.13%) |
| Personal loss to Covid | 99/449 (22.05%) | 99/449 (22.05%) | 98/443 (22.12%) | 93/432 (21.53%) | 93/428 (21.73%) | 87/402 (21.64%) | 79/367 (21.53%) | 79/357 (22.13%) |
| Medical cond. inc. risk | 180/438 (41.1%) | 180/438 (41.1%) | 176/432 (40.74%) | 174/422 (41.23%) | 174/419 (41.53%) | 159/391 (40.66%) | 147/360 (40.83%) | 144/350 (41.14%) |
| Complete CROS | 14.21±8.36 | 14.21±8.36 | 14.21±8.36 | 14.34±8.27 | 14.19±8.23 | 14.20±8.30 | 14.10±8.30 | 14.17±8.28 |
| F1: Volume | 0.73±0.98 | 0.73±0.98 | 0.73±0.98 | 0.74±0.99 | 0.72±0.97 | 0.72±0.98 | 0.70±0.96 | 0.71±0.97 |
| F2: Demoralization | 0.81±0.93 | 0.81±0.93 | 0.81±0.93 | 0.82±0.93 | 0.81±0.93 | 0.82±0.92 | 0.82±0.93 | 0.80±0.92 |
| F3: Risk | 1.76±0.99 | 1.76±0.99 | 1.76±0.99 | 1.77±0.97 | 1.75±0.98 | 1.77±0.97 | 1.76±0.96 | 1.77±0.96 |
| Prior traumas | 0.47±0.80 | 0.48±0.81* | 0.53±0.83*** | 0.55±0.84*** | 0.56±0.85*** | 0.59±0.86*** | 0.63±0.87*** | 0.65±0.89*** |
| Complete PCL5 | 27.67±18.35 | 27.67±18.35 | 27.66±18.44 | 27.77±18.35 | 27.74±18.45 | 27.67±18.35 | 27.19±18.44 | 27.32±18.39 |
| Complete PHQ9 | 9.87±6.59 | 9.87±6.59 | 9.86±6.61 | 9.91±6.58 | 9.89±6.61 | 9.90±6.60 | 9.87±6.61 | 9.87±6.59 |
| Complete ISI | 12.26±5.60 | 12.26±5.60 | 12.19±5.59 | 12.22±5.56 | 12.22±5.56 | 12.13±5.58 | 12.08±5.52 | 12.10±5.54 |
| Complete GAD7 | 9.29±6.12 | 9.29±6.12 | 9.27±6.12 | 9.32±6.11 | 9.26±6.11 | 9.32±6.12 | 9.29±6.12 | 9.23±6.10 |
| PHQ9 item 9 | 0.23±0.61 | 0.23±0.61 | 0.23±0.61 | 0.23±0.61 | 0.23±0.61 | 0.23±0.61 | 0.23±0.61 | 0.23±0.61 |
| Complete work assessment | 3.11±1.99 | 3.11±1.99 | 3.10±1.99 | 3.11±1.99 | 3.09±1.98 | 3.08±1.96 | 3.05±1.94 | 3.05±1.91 |
| Complete occupational retention assessment | 3.61±1.81 | 3.61±1.81 | 3.60±1.82 | 3.59±1.81* | 3.61±1.81 | 3.59±1.80 | 3.68±1.82 | 3.66±1.81 |

Supplementary Figures:


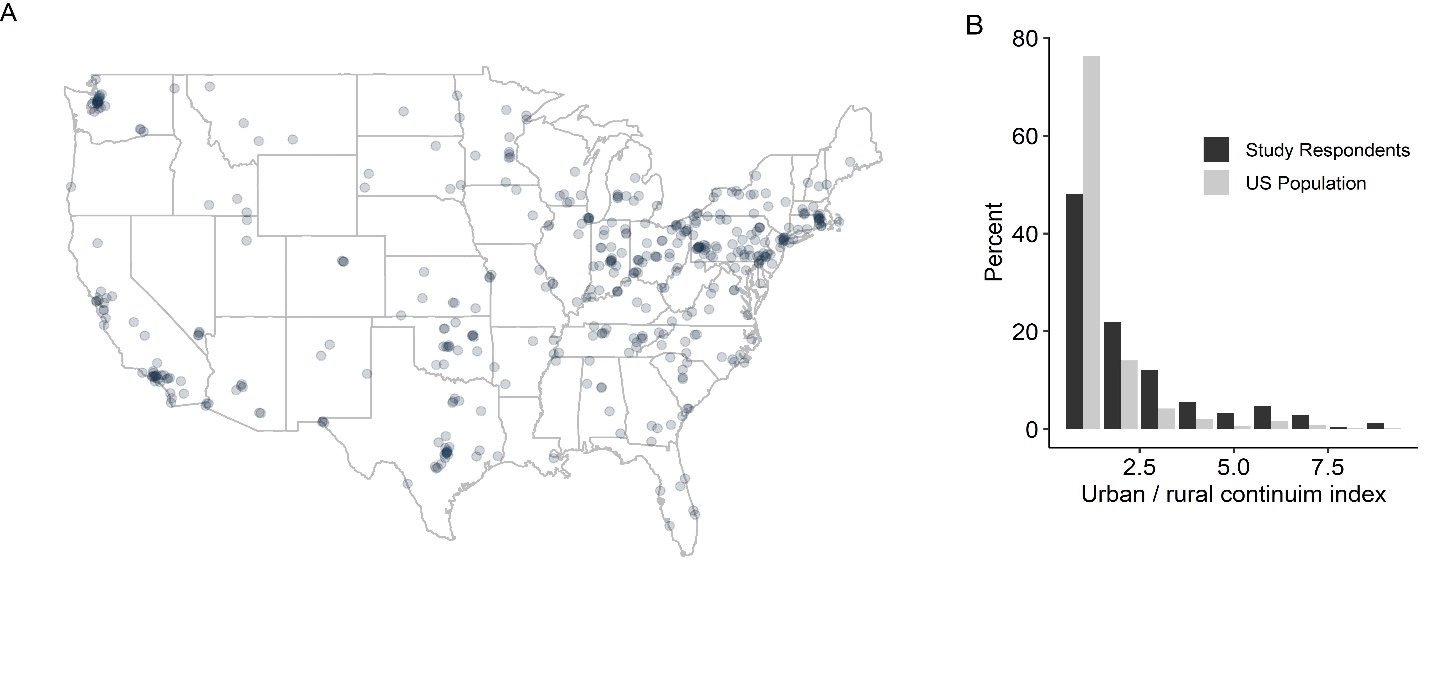


**Supplementary Figure 1. Geographic distributions of respondents.** Responses were obtained from 47 states (A) and 445 zip codes. The distribution of respondents across the USDA Economic Research Service defined Rural-Urban Continuum Codes was similar to that of the US population as a whole, but with some relative over-representation of more rural areas relative to urban areas (B).


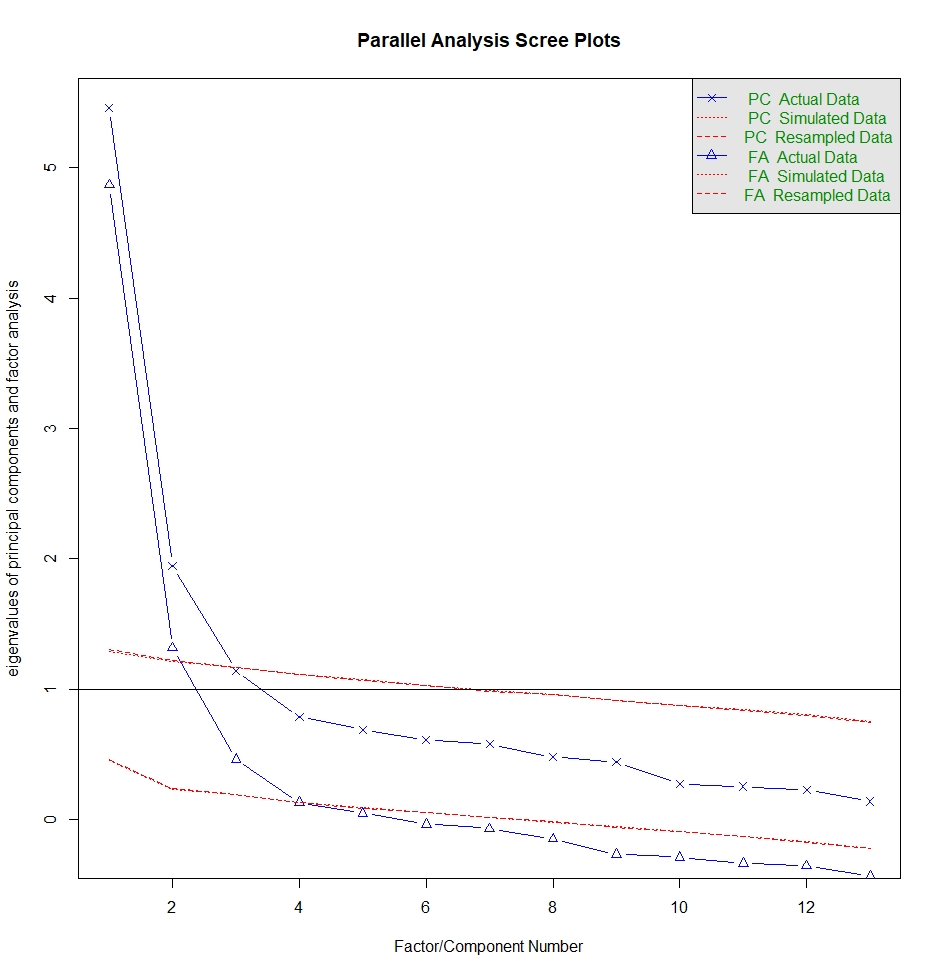


**Supplemental Figure 2**. Scree plot to determine factor number for COVID-19 related occupational stressor (CROS) factorization analysis.


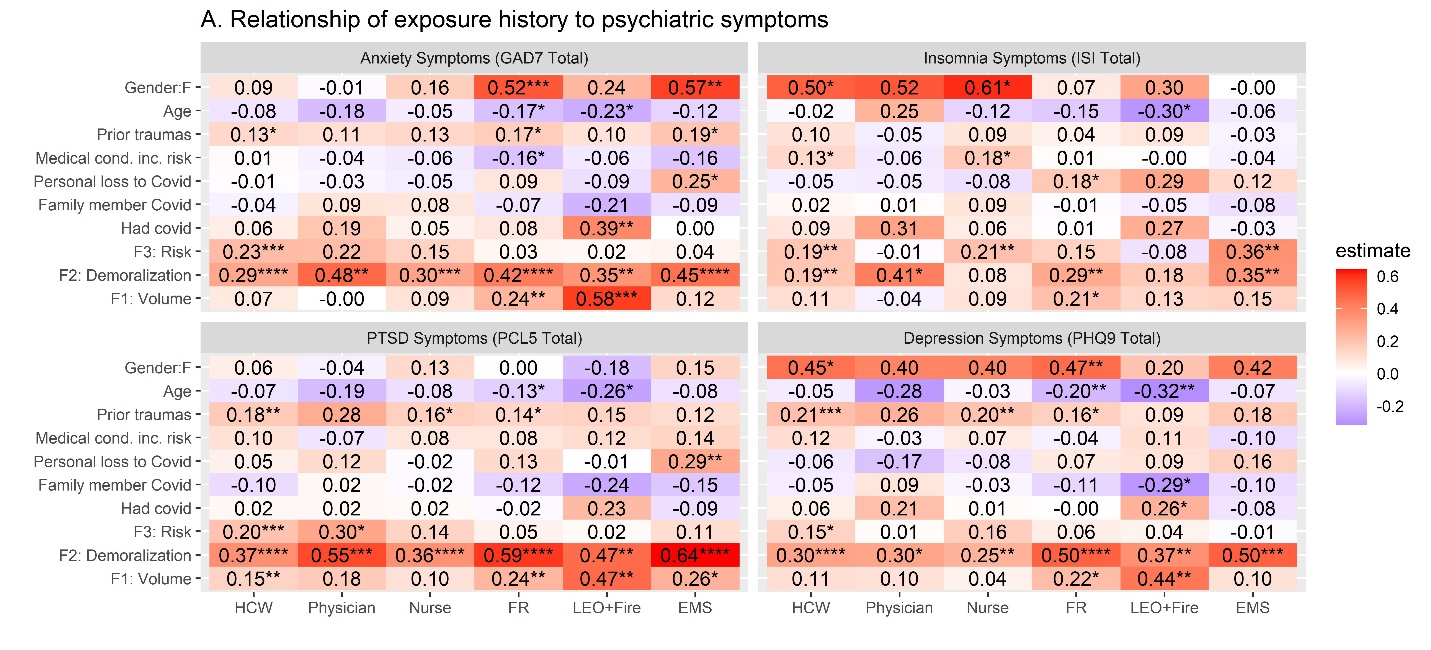


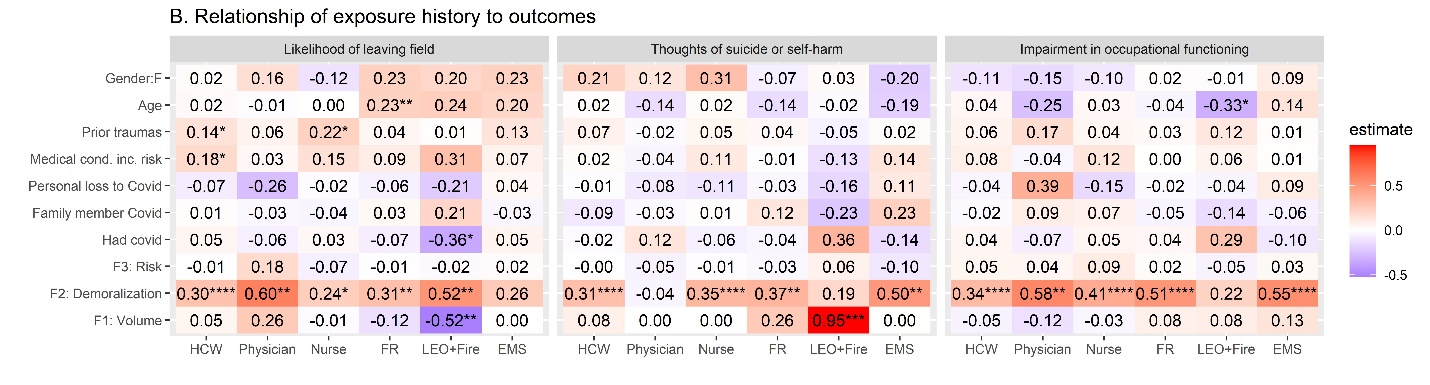


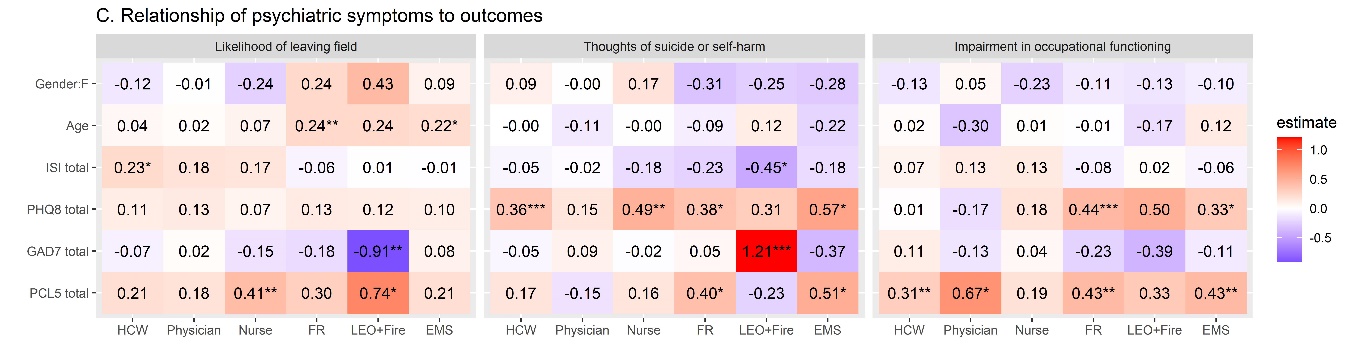


**Supplemental Figure 3. Relationships between different CROS factors, psychiatric symptom expression, and functional outcomes for subgroups.**  Results of multivariable regression models relating CROS factors and covariates to (top row) psychiatric symptom clusters and (middle row) outcome measures. Bottom row: Results of multivariable regression models relating symptom clusters as measured by total scores on the PCL5, PHQ9, GAD7 and ISI, along with covariates of age and gender, to functional outcome measures. Values provided and shading represent β coefficients using normalized predictor variables. *p<.05, **p<.01, ***p<.001, ****p<.0001

**Appendix A: COVID-19 Occupational Stressors (CROS) Assessment for health care workers and first responders**

*1) Binary questions, asked without reference to a specific time period:*

**At any point, have you experienced the following?** *(Yes, No)*

1. Had a known or strongly suspected infection with COVID-19
2. Had a family member that you live with or see regularly had a known or strongly suspected infection with COVID-19
3. Experienced the death of family member or close colleague from known or suspected COVID-19
4. **Do you have medical conditions (not age alone) that put you at higher risk from COVID-19?** *(Yes, No)*

*2) Likert scale questions, asked with reference to the past two weeks*

**In the following questions, we will ask about your work-related experiences with individuals known or strongly suspected to have COVID-19.**Please apply these questions to your own work experiences as a health care worker or first responder. For example, if a question asks about "caring for patients" and you are a first responder, please include any experiences you have responding to calls, even if you would not normally use the word "patient".

**How often did you experience the following:**

*(0=Not at all, Several Days, More than half the days, 3=Nearly every day)*

1. Caring for someone with mild symptoms related to COVID-19
2. Caring for someone critically ill with COVID-19
3. Working longer hours than usual in order to provide assistance or care to individuals with COVID-19
4. Witnessing or responding to a death related to COVID-19, or losing a patient you had been caring for to COVID-19
5. Caring for patients who have died without family physically present due to COVID-19 precautions
6. Caring for patients with known or suspected COVID-19 when you were not able to utilize appropriate PPE for the situation
7. Feeling your care for patients with COVID-19 has been futile or unhelpful
8. Feeling unable to provide high quality care to all patients
9. Feeling at increased risk for contracting COVID-19 due to your work
10. Feeling your family was at increased risk for contracting COVID-19 due to your work
11. Feeling unsupported by your workplace
12. Maintaining separation from a member of your household in order to reduce the risk to those you live with
13. Being expected to do things professionally that unnecessarily increase your risk of exposure to COVID-19?
